# Supplementary material for: Visualizing subcellular changes in the NAD(H) pool size versus redox state using fluorescence lifetime imaging microscopy of NADH
Source: Commun Biol. 2024 Apr 9;7:428. doi: 10.1038/s42003-024-06123-7 (PMC11004000; doi:10.1038/s42003-024-06123-7)
Supplement: Supplementary file 2 — Supplementary Information [file 42003_2024_6123_MOESM2_ESM.pdf]

Supplementary Figures

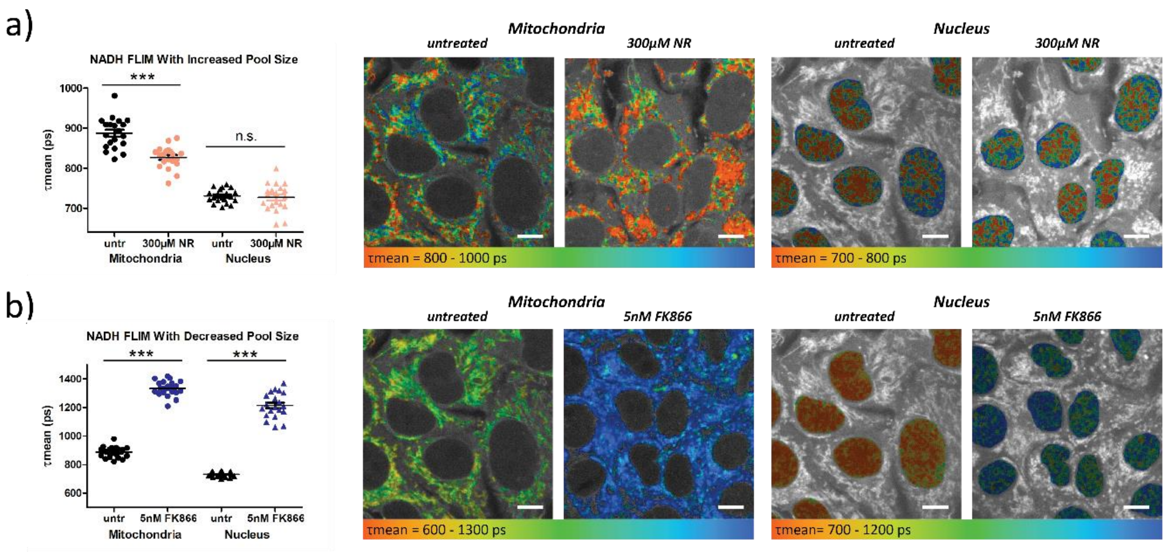

Figure S1. The impact of NAD(H) pool size on NADH FLIM lifetime is consistent across cell types.
a) Mean NADH lifetime (τmean) of mitochondria and nuclei in untreated and nicotinamide riboside (NR) treated HEK293 cells (n = 20 for both) with exemplary images encoding τmean in false-colors (red = shorter, blue = longer). Scale bars represent 10 microns. **b)** Mean NADH lifetime (τmean) of mitochondria and nuclei in untreated and FK866 treated HEK293 cells (n = 20 for both) with exemplary images encoding τmean in false-colors (red = shorter, blue = longer). Scale bars represent 10 microns. Each data point is independent. The bars indicate the mean and standard error. Significances were calculated using t tests between selected groups and are indicated as n.s. for not significant and *** for $p < 0.0001$.

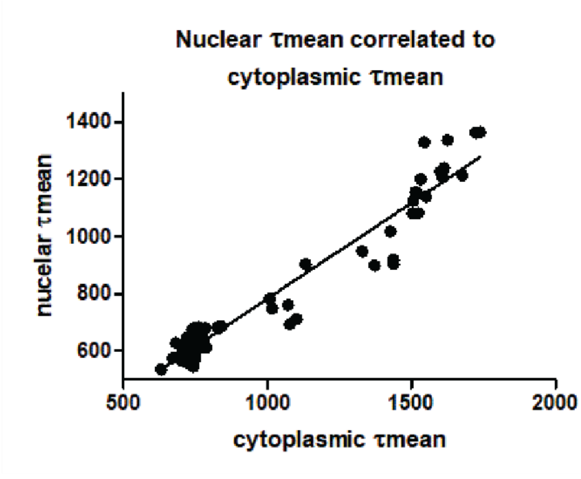

Figure S2. Cytoplasmic and nuclear τmean are closely correlated.
Cytoplasmic τmean correlated with nuclear in untreated, NR treated, and FK866 treated 143B cells. Each data point represents analysis from a single image with τmean averaged across 5 cells (n = 75).

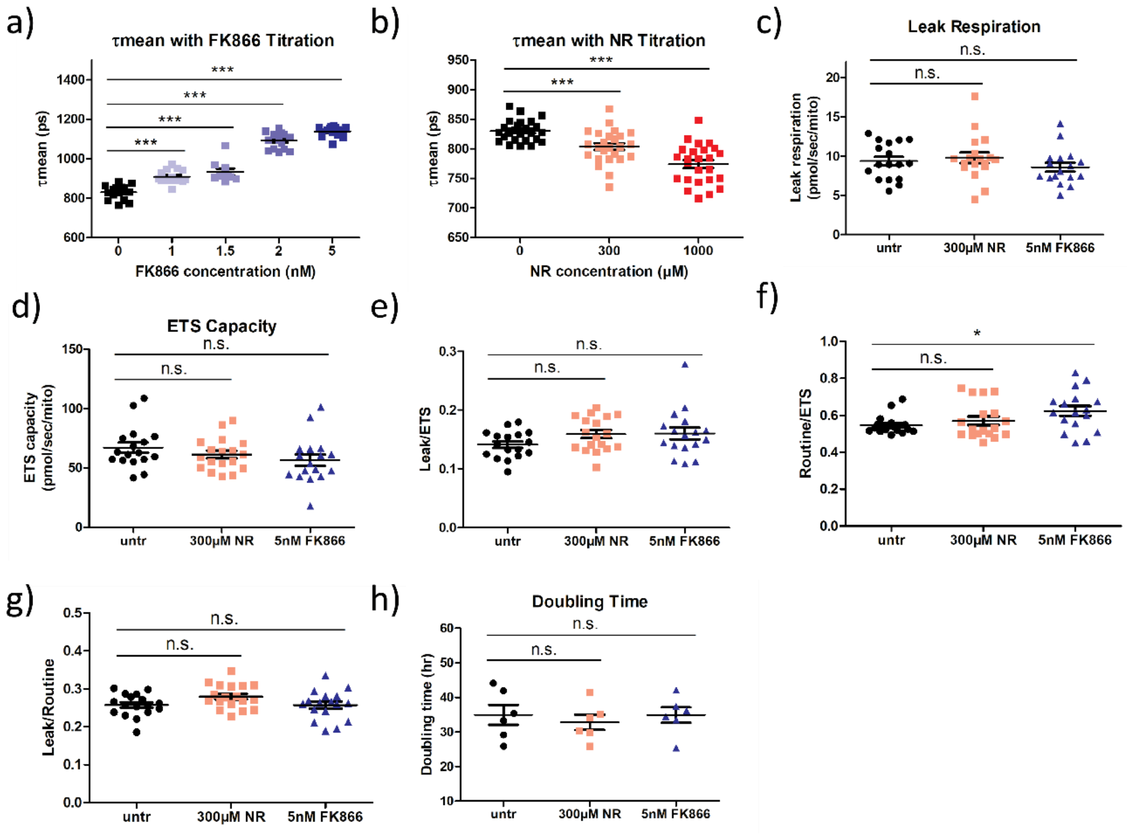

Figure S3. Additional parameters confirm differences in NADH FLIM following pool size modifications without changes in respiration.

a) Mean NADH lifetime (τmean) of mitochondria in HEK293 cells upon 24h treatment with different concentrations of nicotinamide riboside (NR) (n = 25 for all groups). **b)** Mean NADH lifetime (τmean) of mitochondria in HEK293 cells upon 24h treatment with different concentrations of FK866 (n = 15 for 0, 1, 2 and 5 nM FK866, n = 10 for 1.5 nM FK866). **c)** Leak respiration of intact untreated HEK293 cells (n = 17), NR treated HEK293 cells (n = 18), and FK866 treated HEK293 cells (n = 17). **d)** Electron transfer pathway (ETS) capacity of intact untreated HEK293 cells (n = 17), NR treated HEK293 cells (n = 18), and FK866 treated HEK293 cells (n = 17). **e/f/g)** Respiratory ratios of intact untreated HEK293 cells (n = 17), NR treated HEK293 cells (n = 18), and FK866 treated HEK293 cells (n = 17). **h)** Doubling time of untreated HEK293 cells and HEK293 cells treated with 300µM NR or 5nM FK866 (n = 6 for all groups). Each data point is independent. The bars indicate the mean and standard error. Significances were calculated using t tests between selected groups for (a-b) and using ANOVA and Dunn's post hoc test between selected groups for (c-h) and are indicated as n.s. for not significant and *** for p < 0.001.

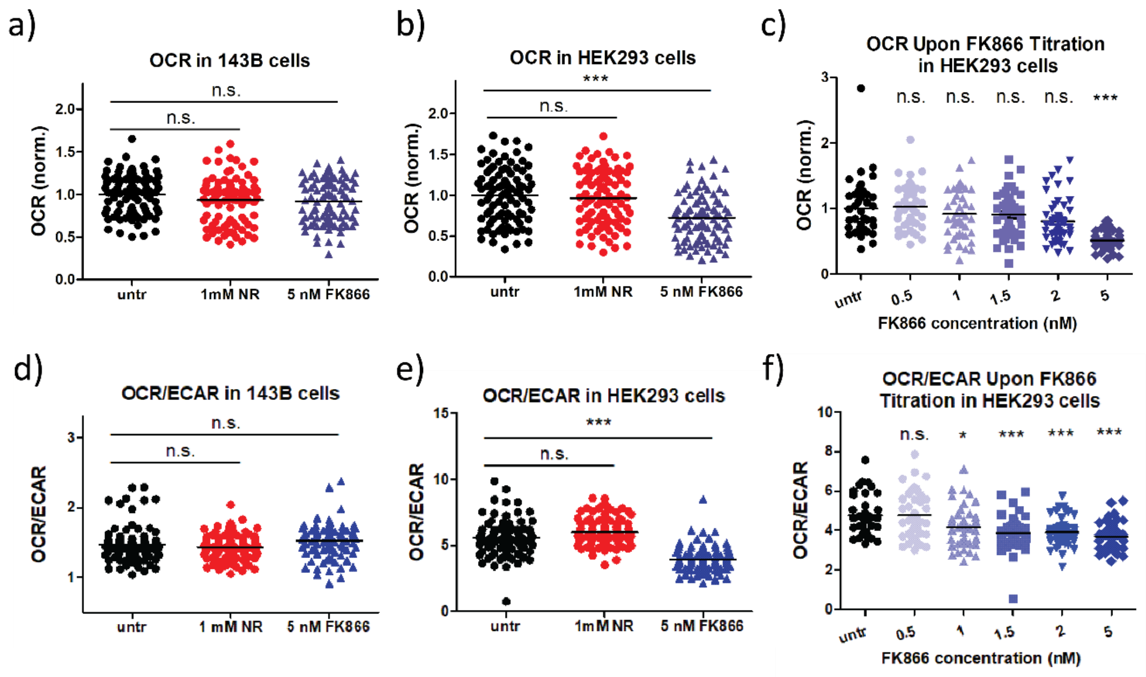

Figure S4. Seahorse assays show that lifetime changes upon NR and FK866 treatment are not due to changes in metabolism.

a) Baseline OCR as measured by Seahorse assay in untreated 143B cells (n = 90), NR treated 143B cells (n = 94), and FK866 treated 143B cells (n = 92). **b)** Baseline OCR as measured by Seahorse assay in untreated HEK293 cells (n = 90), NR treated HEK293 cells (n = 94), and FK866 treated HEK293 cells (n = 92). **c)** Baseline OCR as measured by Seahorse assay in HEK293 cells with FK866 titration (n = 42 for untr and 5nM FK866, n = 48 for 0.1, 1, 1.5, and 2 nM FK866). **d)** Baseline OCR/ECAR as measured by Seahorse assay in in untreated 143B cells (n = 90), NR treated 143B cells (n = 94), and FK866 treated 143B cells (n = 92). **e)** Baseline OCR/ECAR as measured by Seahorse assay in in untreated HEK293 cells (n = 90), NR treated HEK293 cells (n = 94), and FK866 treated HEK293 cells (n = 92). **f)** Baseline OCR/ECAR as measured by Seahorse assay in HEK293 cells with FK866 titration (n = 42 for untr and 5nM FK866, n = 48 for 0.1, 1, 1.5, and 2 nM FK866). n = 3 independent experiments for all Seahorse assays. Each data point is a biologically independent sample. The bars indicate the mean and standard error. Significances were calculated using ANOVA and Dunn's post hoc test between selected groups and are indicated as n.s. for not significant, * for $p < 0.05$, ** for $p < 0.01$, and *** for $p < 0.001$.

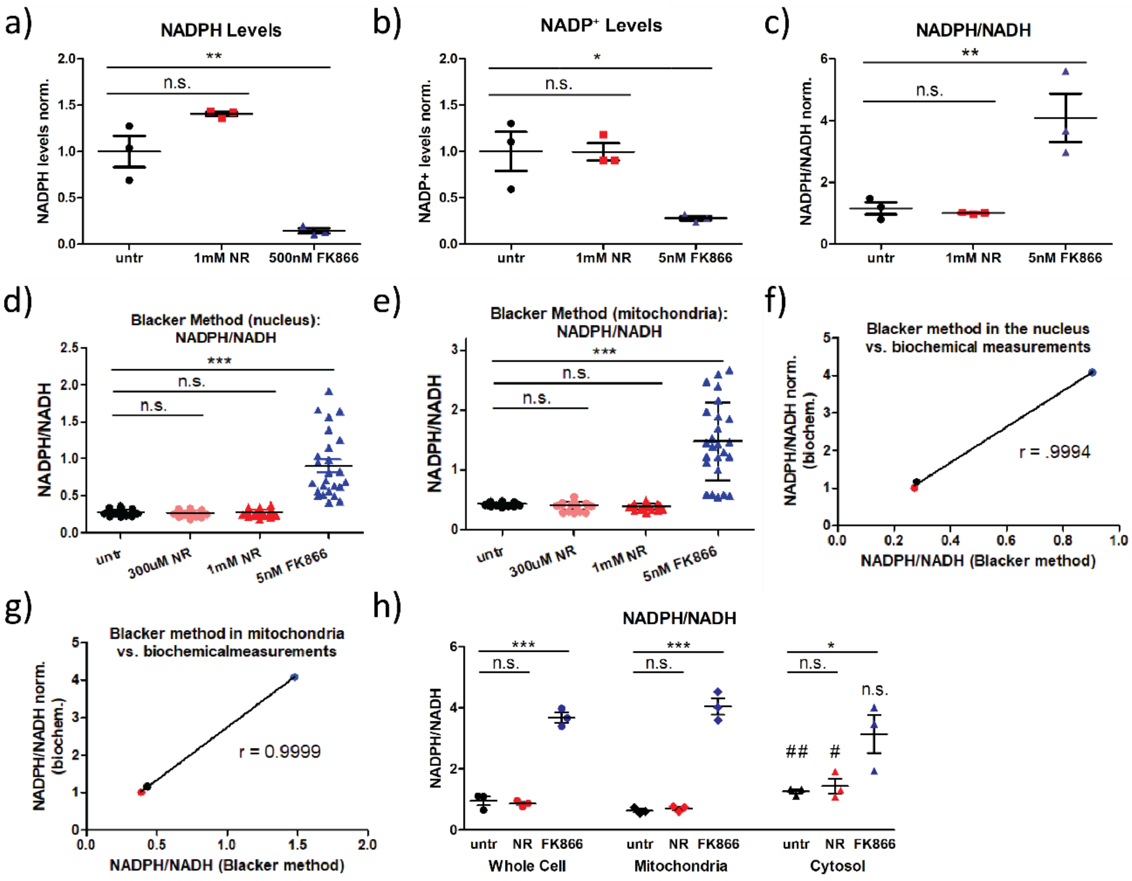

Figure S5. NADP(H) pool is similarly altered as NAD(H) pool by NR and FK866 treatments.

a/b/c) NADPH levels (a), $NADP^+$ levels (b), and NADPH/NADH ratio (c) quantified biochemically in HEK293 cells upon 24h treatment with nicotinamide riboside (NR) or FK866 (n = 3 for all groups). **d/e)** NADPH/NADH ratio in the nucleus (d) and the mitochondria (e) quantified using FLIM parameters by Blacker et al.'s methods [15] (n = 25 for all groups). **f/g)** Correlation between average NADPH/NADH as quantified using Blacker et al.'s method [15] in the nucleus (f) and the mitochondria (g) and biochemically quantified average NADPH/NADH (as shown in (c)). Correlations were calculated using linear regression. **h)** NADPH/NADH ratio quantified biochemically upon 24 hour treatments of 5nM FK866 and 1mM NR in whole cells and in mitochondria and cytosol after subcellular fractionation (n = 3 for all groups). Each data point is independent. Significances between untreated and NR/FK866 treated samples are indicated with *, and significances between mitochondrial and cytosol within each treatment group (untr, NR, FK866) are indicated with #. The bars indicate the mean and standard error. Significances were calculated using ANOVA and Dunn's post hoc test between selected groups and are indicated as n.s. for not significant, */# for $p < 0.05$, **/## for $p < 0.01$ and ***/### for $p < 0.001$.

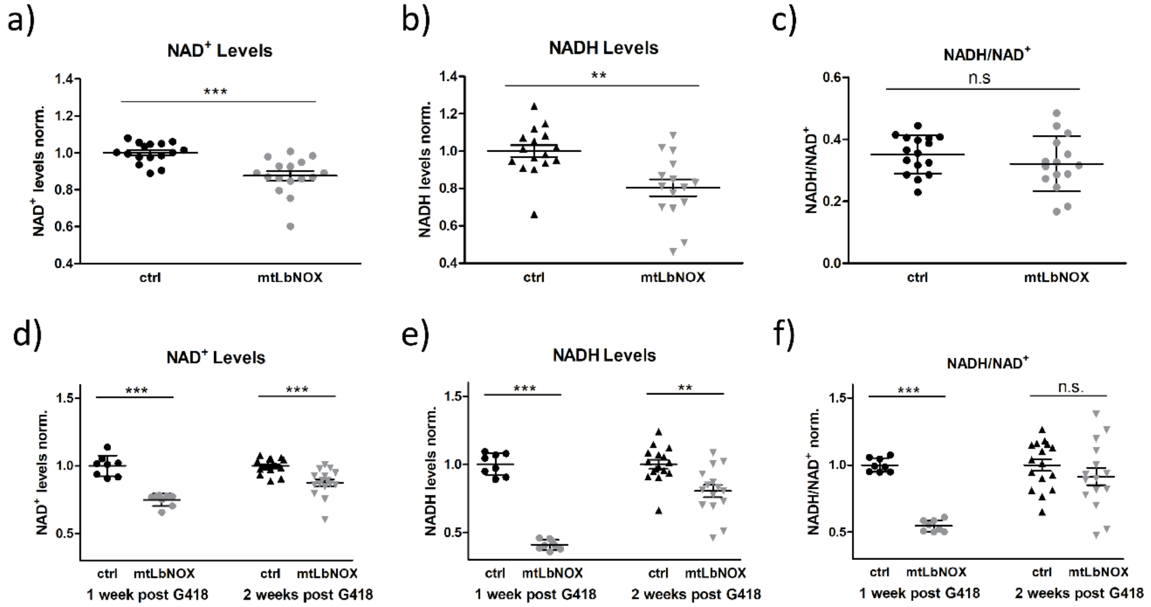

Figure S6. Redox ratio in mt*Lb*NOX expressing cells equilibrates over time.
a) NAD^+ levels quantified biochemically with the NAD/NADH Quantification Kit (Sigma-Aldrich) in 143B control cells and 143B mt*Lb*NOX expressing cells around 2 weeks after starting selection pressure (n = 16 for all groups). **b)** NADH levels quantified biochemically with the NAD/NADH Quantification Kit (Sigma-Aldrich) in 143B control cells and 143B mt*Lb*NOX expressing cells around 2 weeks after starting selection pressure (n = 16 for control, n = 15 for mt*Lb*NOX). **c)** $NADH/NAD^+$ ratio quantified biochemically with the NAD/NADH Quantification Kit (Sigma-Aldrich) in 143B control cells and 143B mt*Lb*NOX expressing cells around 2 weeks after starting selection pressure (n = 16 for control, n = 15 for mt*Lb*NOX). **d-f)** NAD^+ levels (d), NADH levels (e), and $NADH/NAD^+$ ratio (f) quantified biochemically 1 week (n = 8 for all groups) and 2 weeks (data same as a-c) after starting selection pressure. Each data point is independent. The bars indicate the mean and standard error, and significances were calculated using t tests between selected groups and are indicated as n.s. for not significant, ** for $p < 0.01$, and *** for $p < 0.001$.

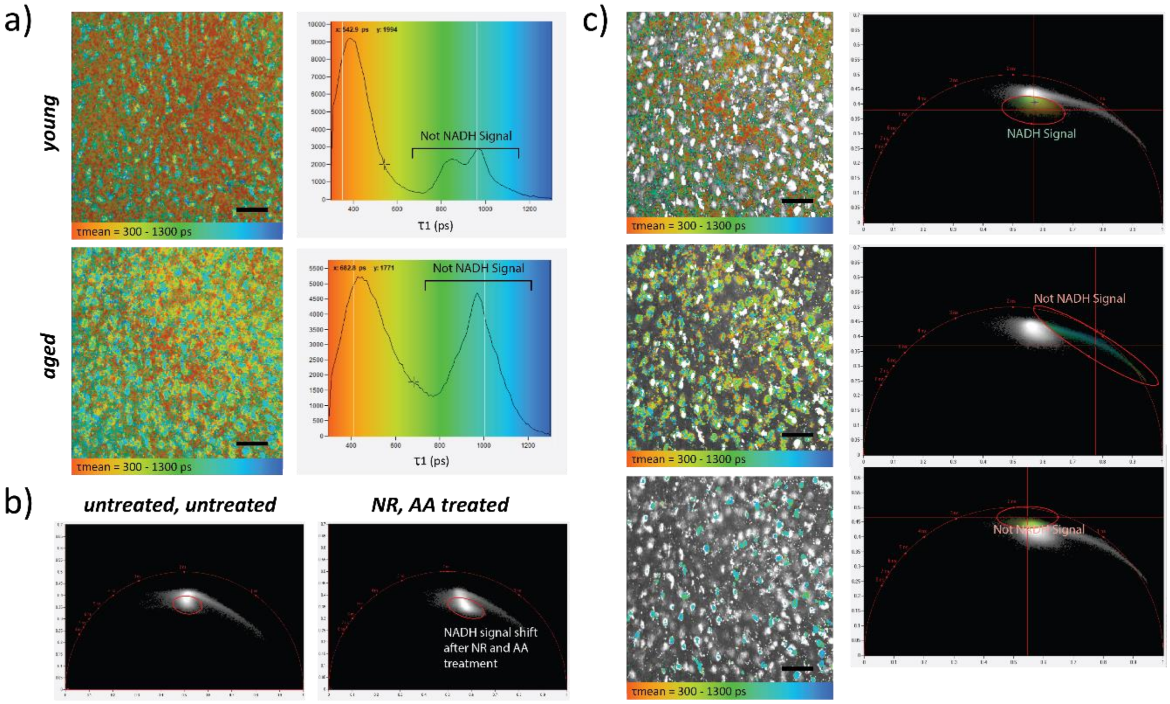

Figure S7. Identifying and separating NADH autofluorescence in mouse liver tissues.
a) Representative FLIM images of liver from young (6 months, top left) and aged (18 months, bottom left) mice which encode τmean in false-colors (red = shorter lifetime, blue = longer lifetime). τ1 distribution for both representative images are shown with non-NADH signal labeled. **b)** Representative phasor plots of liver tissue from saline treated mice untreated with antimycin A (left) and antimycin A treated liver tissue from NR treated mice. NADH signal region is outlined in red. **c)** Representative FLIM images of liver tissue with different regions of the phasor plot selected. Scale bars represent 20 microns.

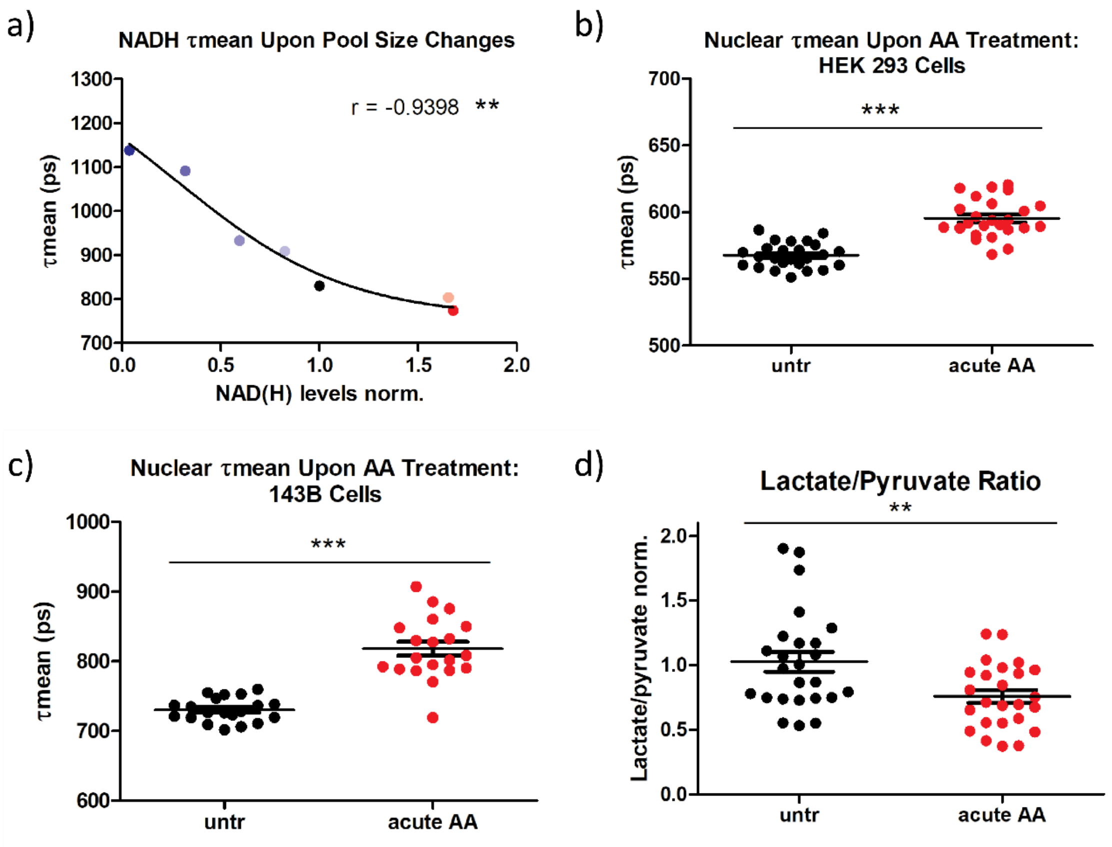

Figure S8. Metabolic delta is an imperfect parameter for isolating alterations in respiration from NADH FLIM.

a) Average mean NADH lifetime (τmean) correlated with the average NAD(H) pool size in HEK293 cells upon 24h treatment with varying concentrations of nicotinamide riboside (NR) or FK866 (more saturated dots represent higher concentrations). Blue dots are FK866 treated and orange-red dots are NR treated. **b)** Mean NADH lifetime (τmean) in untreated HEK293 nuclei and HEK293 nuclei upon acute (5min) treatment with antimycin A (n = 25 for both groups). **c)** Mean NADH lifetime (τmean) in untreated 143B nuclei and 143B nuclei upon acute (5min) treatment with antimycin A (n = 20 for both groups). **d)** Lactate to pyruvate ratio of untreated HEK293 cells and HEK293 cells treated with acute (5min) antimycin A (n = 25 for both groups). Each data point is independent. The bars indicate the mean and standard error. Significances were calculated using t tests between groups and are indicated as ** for $p < 0.01$ and *** for $p < 0.001$.

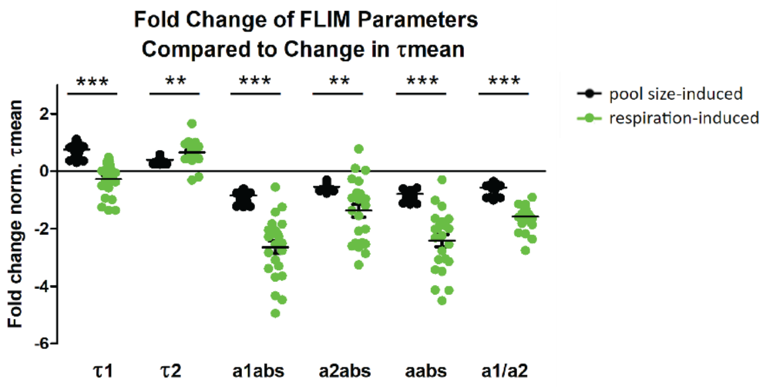

Figure S9. NADH lifetime components allow differentiation between respiration and pool size induced lifetime changes in HEK293 cells.

Fold change of FLIM lifetime components (τ1, τ2, a1abs, a2abs, aabs, and a1/a2) normalized to fold change in τmean between HEK293 cells treated with NR and FK866 (pool size-induced) (n = 25) and between untreated and antimycin A treated cells (respiration-induced) (n = 24). Each data point is independent. The bars indicate the mean and standard error. Significances were calculated using t tests between selected groups and are indicated as ** for p< 0.01 and *** for p < 0.001.
